# Voluntary wheel running alters markers of amyloid-beta precursor protein processing in an ovarian hormone depleted model

**DOI:** 10.3389/fendo.2022.1069404

**Published:** 2022-12-06

**Authors:** Ahmad Mohammad, Michael S. Finch, Jacob Sweezey-Munroe, Rebecca E. K. MacPherson

**Affiliations:** ^1^ Department of Health Sciences, Brock University, St. Catharines, ON, Canada; ^2^ Centre for Neuroscience, Brock University, St. Catharines, ON, Canada

**Keywords:** physical activity, brain derived neurotrophic factor, estrogen, beta-secretase, Alpha-secretase

## Abstract

**Introduction:**

Aberrant cleavage of the transmembrane protein, amyloid-beta precursor protein (ABPP), results in the overproduction of amyloid-beta (AB) peptides which can form senile plaques in the brain. These plaques can get lodged within synapses and disrupt neuronal communication ultimately leading to rampant neuron death. The rate-limiting enzyme in AB production is beta-site ABPP cleaving enzyme 1 (BACE1). In females, estrogen loss is associated with increases in AB and BACE1 content and activity. Exercise is known to have anti-amyloidogenic effects and may be able to alter BACE1 in cases of ovarian hormone depletion. This study aimed to examine the effects of physical activity on BACE1 in intact and ovariectomized female mice.

**Methods:**

Female C57BL/6 mice (24 weeks old) underwent bilateral ovariectomy (OVX; n=20) or SHAM surgery (SHAM; n=20). Mice were assigned to one of four groups (n=10/group) for 8 weeks: (1) sham (SHAM), (2) sham with a wheel (SHAM VWR), (3) ovariectomized (OVX), or (4) ovariectomized with a wheel (OVX VWR).

**Results:**

Novel object recognition testing demonstrated that OVX mice had a lower percentage of novel object investigation time compared to SHAM. OVX mice also had higher prefrontal cortex BACE1 activity compared to SHAM (p<0.0001), while the OVX+VWR activity was not different from SHAM.

**Discussions:**

Our results demonstrate that voluntary wheel running in an ovariectomized model prevented increases in BACE1 activity, maintained memory recall, and may provide a method of slowing the progression of Alzheimer’s disease.

## Introduction

Alzheimer’s disease (AD) is a neurodegenerative disease classified as the most prevalent form of dementia accounting for nearly 60% of cases (1, 2). There are two major classifications of AD, sporadic and familial with sporadic having a later disease onset (>65 years of age) (3, 4). Familial AD accounts for ~ 3-5% of cases and is caused by genetic mutations in presenilin 1, presenilin 2, or amyloid precursor protein (ABPP) (1, 5, 6). Sporadic AD, however, accounts for ~95% of cases, and while there are non-modifiable risk factors such as age, sex, and APOE4 genotype that increase the risk, there are also modifiable factors, such as physical inactivity (1, 3, 5, 6). A non-modifiable risk factor that impacts AD patients most is biological sex, as females represent ~70% of AD cases (3, 5, 7). Initially, it was thought that this was due to the longer lifespan of females, however, even after accounting for age, the difference between males and females is present (8). It is possible that this discrepancy is caused by the estrogen depletion that females experience during menopause (8–12). Estrogen is the most abundant ovarian hormone and is a regulator of brain-derived neurotrophic factor (BDNF), a protein that maintains neuronal plasticity and health. Circulating BDNF is lower in menopausal women (10–14) and may therefore be a contributing factor to the increased rate of AD in females.

One of the primary pathologies of AD is the presence of extraneuronal senile plaques (2–4, 13). These plaques are formed when there are accumulations of amyloid-B (AB) peptides that aggregate in the extracellular matrix of the brain. The plaques can disrupt synaptic function, cell signaling, and lead to neuron death. The aggregation of such plaques begins decades before clinical symptoms manifest and therefore it is important to examine markers related to the production of the peptides as opposed to the plaques themselves (2, 3, 15). AB peptides are formed by the aberrant cleavage of the transmembrane protein, ABPP. The cleavage of ABPP can occur through two major pathways (4, 6–10, 13, 15). The first pathway is non-amyloidogenic and is initiated by α-secretase (ADAM10). When ADAM10 initiates ABPP processing, it cleaves ABPP within the AB domain, resulting in no AB peptide production and the subsequent production of non-pathological proteins. In the amyloidogenic pathway, however, ABPP is cleaved by B-site amyloid precursor protein cleaving enzyme 1 (BACE1) (16–19). BACE1 is the rate-limiting step in AB peptide production and a marker of amyloidogenic activity. BACE1 activity and content as well as its downstream effects are elevated in cases of menopause as well as ovarian hormone loss in animal models of ovariectomy (20–22). Rodent models of menopause/ovarian hormone loss also demonstrate elevated BACE1 content and activity as well as a higher concentration of AB peptides and subsequent plaques suggesting an underlying relationship between these mechanisms (11, 20, 21, 23–25).

There is an inverse relationship between BDNF and BACE1 content and activity (2, 5, 13, 26). The connecting mechanism between the two proteins has yet to be fully elucidated, however, patients with AD tend to have lower circulating and brain concentrations of BDNF compared to healthy controls (3, 5, 7). In support of a relationship between BDNF and BACE1, direct treatment of brain sections with BDNF results in reduced BACE1 activity (3, 11, 13). Further, higher BDNF content correlates with elevations in ADAM10 content and activity (3, 4, 7, 26) and treatment of SH-SY5Y neuronal cells with BDNF increases ADAM10 activity (7). These relationships indicate that BDNF can manipulate ABPP processing, pushing it towards the non-amyloidogenic pathway and away from the amyloidogenic pathway (4, 6, 13, 23). Although neuronal BDNF content can vary between sexes, as one ages, there is an expected decrease regardless of sex (1, 3, 23). Certain factors can exacerbate this decline, for example, post-menopausal females and ovariectomized rodent models have been shown to have lower levels of BDNF than age-matched male counterparts or even pre-menopausal females (27, 28). The connection between estrogen and BDNF is further supported by the fact that there is an estrogen-sensitive response element on the BDNF gene (7, 9, 20, 24). This demonstrates a mechanistic relationship between estrogen signaling and BDNF further connecting the pathways.

The activation of the transcription factor c-AMP response element-binding protein (CREB) is an important regulator of BDNF gene expression and is downstream of both estrogen (8–10, 12) and BDNF signaling (3, 5, 8, 9, 14). In models of estrogen loss such as an ovariectomized (OVX) mouse model, BDNF levels tend to be reduced indicating that estrogen plays a role in the expression of BDNF (9, 10). It is also believed that these reductions in BDNF content with the loss of estrogen contribute to declines in cognition and memory (8, 9, 11). This relationship between estrogen and BDNF may explain why estrogen depletion is commonly accompanied by an increase in BACE1 activity and ABPP processing.

The main purpose of the current study is to determine if exercise in the absence of ovarian hormones in a mouse model will increase BDNF and decrease BACE1 activity. Based on previous literature, it is expected that the loss of estrogen in OVX mice will increase BACE1 activity and reduce BDNF levels. However, we hypothesize that voluntary exercise will blunt and prevent reductions in BDNF and increases in BACE1 activity in an OVX model. We therefore aim to introduce novel insight into the relationships between ABPP processing, estrogen signaling, and BDNF as well as any other beneficial effects from voluntary exercise.

## Methods

### Animals and design

Forty female C57BL6/J mice arrived at the laboratory at 28 weeks of age (Jackson Laboratory, Bar Harbor, ME). Before arriving at Brock University, half the mice underwent a bilateral ovariectomy (OVX=20) while the other half underwent a sham surgery (SHAM=20) when they were 24 weeks of age while still at Jackson Laboratory (11). OVX is the standard model of inducing estrogen depletion in rodent models as the ovaries are responsible for most endogenous estrogen production (8, 9, 11). The mice were then acclimated for 7 days before they were housed in pairs in the Techniplast DV80 caging rack for the extent of the study (8 weeks). The Techniplast Digital Ventilated Cage 80 (DVC80) system is equipped with GYM500 software that allows for 24/7 monitoring of cage ambulation and voluntary wheel running (VWR). Half of the mice were provided with a cage wheel allowing them *ad libitum* access to wheel running, whereas the other half were not provided with a wheel and were classified as sedentary (OVX=10; SHAM=10). The DV80 caging system is equipped with activity monitoring software to track cage wheel activity (time and distance 24/7) over the course of the study (OVX VWR= 10; SHAM VWR=10). All the mice had *ad libitum* access to food and water and were housed at room temperature. All mice followed a standard 12-h light/dark schedule with body mass and food intake measured weekly at the beginning of a light cycle. All experimental procedures were pre-approved by the Brock University Animal Care Committee (AUP: No. 17-12-01) and followed all guidelines of the Canadian Council on Animal Care.

### Novel object recognition testing

A novel object recognition test (29–32) was performed in the week leading up to endpoint. Prior to the beginning of testing, all procedures were adapted and in accordance with the standard guidelines and operating procedures of Brock University. The NORT was performed in four individual steps which involved two habituation phases, one familiarization phase, and one testing period. For all the required steps, the same open arena was used. The two habituation phases were run 24 hours apart and they involve placing the mice into the same corner of the empty arena and allowing them to explore freely for 10 minutes. After the second habituation period, the mice were left to rest for another 24 hours before the familiarization process (29). This involved placing two of the same objects in the arena with one in the top right and the other in the top left. The objects were placed 5 cm away from both walls they were closest to, before the mice were placed into the arenas again for 10 minutes. The familiarization was recorded, and the arenas were sanitized between trials to remove any odours. The mice were then returned to their home cages for the span of 1 hour. During this time, one of the objects within the arenas was replaced with one that would be novel to the mice implying they had not been exposed to it previously. Once the hour was complete, the testing stage began, and the mice were allowed to freely explore the arena now containing one familiar and one novel object for a span of 10 minutes while being recorded. The arenas were cleaned using virox solution and the testing was repeated until all the mice had been tested after being familiarized. They were given 72 hours to rest before the entire process was repeated with a gap period of four hours instead of one. The second time around, both the familiar and novel objects were changed entirely to ensure that the mice were familiarized with an entirely new object and had a unique novel object as well (11, 29, 30). The NORT was analyzed by calculating the ratio between the time spent investigating the novel object to the total amount of time they spent investigating both objects. A higher value for the ratio indicates that the mice were able to actively remember the familiar object more than the novel one.

### Behavioural phenotyping

Also in the final week of the study, mice were placed in Sable System Promethion High-Definition Behavioural Phenotyping cages (Sable Systems International, Las Vegas, NV) on a 12-hour light/dark cycle for a span of 48 hours (33–35). This system allows for real-time behavior analysis of mice in a home-cage setting. Individual cages (interior dimensions of 31.5 × 15.5 (floor), up to 34.5 × 19.0 (ceiling) × 13.0 cm tall) include a ceiling-mounted food hopper and water bottle. Cages include a ceiling-mounted small “house” into which mice can climb. X- and Y-axis (horizontal plane) photoelectric beam motion detectors are positioned around the cage. While in the cages, any mice who had wheels in their housing cages had wheels installed during their time in the cages which they had *ad libitum* access to. Mice also had *ad libitum* access to food and water as well as a housing unit all of which had mass monitors to measure any changes. Behavioural phenotyping was analyzed based on movement patterns, behaviours (interactions with cage elements), resting time, fine movement, and overall movement were recorded and averaged into 30-minute intervals using Sable Systems data acquisition software (36, 37). Data were analyzed using Sable Systems International Macro Interpreter software (v.2.48) using One-Click Macro.

### Tissue collection

Mice were anaesthetized with a weight adjusted bolus intraperitoneal injection of sodium pentobarbital (5 mg/100 g body mass) before being euthanized *via* exsanguination through blood draw directly from the left ventricle. Blood was allowed to clot at room temperature for ~30 min prior to centrifugation (1500 RCF while at a temperature of 4 °C for 10 minutes) and collection of serum. Following this, the brains were quickly removed and samples from both the right and left hippocampus and prefrontal cortex were isolated and snap frozen in liquid nitrogen. To confirm the loss of ovarian hormones, uterine weights were collected. All isolated tissue was stored in a -80 °C freezer until analysis.

### Western blotting

Both isolated parts of the brain (hippocampus and prefrontal cortex) were weighed and homogenized in 1mg:20µL volumes of NP40 Cell Lysis Buffer (Cat. No. FNN0021, ThermoFisher) containing 50µL of a protease inhibitor (Cat. No. P2714, Sigma-Aldrich) and 34µL of phenylmethylsufonyl fluoride (Cat. No. P7626, Sigma-Aldrich). After this, samples were centrifuged at a temperature of 4 °C for 15 mins at 10,000g before the supernatant was collected. Protein concentrations were determined using a Bicinchoninic Acid (BCA) assay. Western blot samples were prepared using 2x Laemmli buffer and an equalized protein concentration of 1µg/µL. 15 µL of prepared sample was then loaded into each well on a homemade 12.5% SDS-PAGE gel and proteins were electrophoretically separated at 120V for a span of 90 minutes. Proteins were wet transferred from the gel to 0.45 nitrocellulose membranes (Cat. No. 10600002, Cytvia) at 100V for 60 minutes over ice. Following the wet transfer, the membranes were blocked using 5% non-fat dry milk-TBST (Tris-buffered saline/0.1% Tween 20) for 60 minutes at room temperature. Membranes were then incubated at 4 °C with slight agitation overnight in a solution containing the appropriate primary antibody diluted 1:1000 in 5% BSA (bovine serum albumin)-TBST. The next day, membranes were rinsed three times for a total of 15 minutes in TBST after which they were incubated in the appropriate secondary antibody diluted to 1:2000 in 1% non-fat dry milk-TBST for 60 minutes. Membranes were again washed in TBST three times for a total of 15 minutes before proteins were visualized using Western Lightning Plus-ECL (Cat. No. NEL104001EA, PerkinElmer) in a Bio-Rad ChemiDoc Imaging System running Image Lab Touch Software. After the fluorescence images were taken, the membranes were placed in a ponceau stain (Cat. No. PON002, BIOSHOP) for 10 minutes before being rinsed in distilled water and laid out to dry for imaging. The bands of protein were quantified and analyzed using AlphaView followed by a quantification of the ponceau to ensure equal loading across the membrane (<10% variability) (3, 11, 13). Western blotting was used to determine protein marker content of the amyloidogenic proteins BACE1 (Cat. No. 5606S, Cell Signaling), ADAM10 (Cat. No. ab1997, abcam), ABPP (Cat. No. 825001, BioLegend), sABPPα (Cat. No. 11088, IBL) and sABPPB (Cat. No. 813401, BioLegend) as well as markers of BDNF signaling such as pro/mature BDNF (Cat. No. ab108319, abcam), TrkB (Cat. No. 80E3, Cell Signalling), pTrkB (Cat. No. ab109684, abcam), CREB (Cat. No. 48H2, Cell Signaling), Erα (Cat. No. sc-528195, Santa Cruz Biotechnology), ErB (Cat. No. sc-530103, Santa Cruz Biotechnology) and pCREB (Cat. No. S133, Cell Signaling). When analyzing all proteins, the content was made relative to their appropriate ponceau loading control and all phosphorylated proteins were made relative to the total protein content.

### BACE activity assay

Homogenates were made at a concentration of 0.5 µg/µL using extraction buffer. All of the assays were then loaded and used according to the instructions provided by the manufacturer (Cat. No. ab65357, abcam). Fifty µL of prepared prefrontal cortex and hippocampus sample were loaded into a black 96-well plate. While the plate was being loaded, the reaction buffer and all the substrates were preincubated at 37 °C. Once they reached the desired temperature and the plate was loaded, 50 µL of 2x reaction buffer was added to each well followed by 2µL of the BACE substrate. The plate was wrapped in aluminium foil and placed in the dark at 37 °C for 60 minutes after which the plate was read using a Spectra Max M2 plate reader at wavelengths of 335 and 496nm. BACE activity was measured at endpoint and provided as relative florescent units. The florescent unit corresponds directly to the amount of BACE activity there is present within the sample.

### ADAM10 activity assay

ADAM10 activity was detected according to the manufacturer’s instructions using the SensoLyte ADAM10 Fluorometric Activity Assay Kit (Cat. No. AS-72226, SensoLyte). 50 µL of homogenized hippocampus or prefrontal cortex at 0.5 µg/µL of total protein was loaded into a black 96-well plate. Next, 50 µL of diluted ADAM10 substrate was added to each well, and reagents were agitated for 30 seconds by hand. The plate was covered in aluminum foil and placed in the incubator at 37 °C for 60 minutes in the dark. After incubation, a Spectra Max M2 plate reader was used to read fluorescence at endpoint at wavelengths of 490 and 520 nm.

### ELISA

The content of serum BDNF was determined using a commercially available ELISA kit (Cat. No. CYT306, ChemiKine).

### Statistical analysis

SigmaStat and Graphpad Prism 8/9 were used for all statistical analysis. Differences in weekly body mass were assessed using a two-way mixed repeated measures ANOVA. Differences in NORT, uterine tissue weight, all activity assays, and all protein markers were analyzed using a two or three way ANOVA. Any significant interactions were followed up with a Tukey *post hoc* analysis while a Shapiro-Wilk test was used to test normality. Any values that deviated from the mean by two standard deviations were considered outliers and removed from analysis. All significant data was reported as P ≤ 0.05 and data was presented as mean ± standard deviation.

## Results

### Animal model characteristics

**Figure 1:**
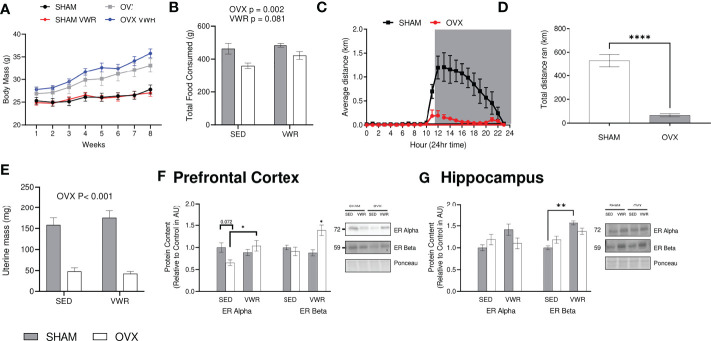
Model characteristics and confirmation of estrogen loss: **(A)** Weekly body mass during the study time (g). **(B)** Total food intake/cage (g). **(C)** Average distance ran (km) per hour. **(D)** Total distance ran voluntarily throughout entire study (km). **(E)** Uterine weight (g). **(F)** Prefrontal Cortex ER Alpha and ER Beta content. **(G)** Hippocampus ER Alpha and Er Beta content. All graphs are accompanied by representative blots. Data analyzed using a two-way ANOVA. ER Alpha, estrogen receptor alpha; ER Beta, estrogen receptor beta; SED, sedentary; VWR, voluntary wheel running; OVX, ovariectomized; SHAM, sham operated. Significance is set to *P ≤ 0.05; **P ≤ 0.005; ***P ≤ 0.0005; P ≤ 0.05; n=10 per group.

At baseline there were no significant differences in body mass (g) across all groups (SHAM=25.3 ± 2.4, OVX=26.9 ± 4.0, SHAM VWR=24.9 ± 1.8, OVX VWR=27.8 ± 1.7, p > 0.05). Body mass increased over the 8 weeks in all groups (p<0.0001), and the SHAM and SHAM VWR groups had a lower body mass (g) compared to OVX and OVX VWR through weeks 1 to 8 (**Figure 1A**; p ≤ 0.05). There were no differences between SHAM and SHAM VWR (p > 0.05) or between OVX and OVX VWR (p > 0.05) at any week showing no effect of exercise and implying there was only a main effect of OVX status (p < 0.05). Final body mass at week 8 indicated a main effect of ovariectomy (p < 0.0001), with no effect of voluntary wheel running (p > 0.05; **Figure 1A**). There was no effect of exercise on food intake (p = 0.081) however, there was a main effect of OVX indicating that the SHAM mice had a higher average food intake in grams per cage (SHAM=474.1 ± 52.4, OVX=397.6 ± 55.8, p = 0.002; **Figure 1B**). Distance run was measured every hour and average distances were taken per hour throughout the study. During the light-cycle, both the sham and ovariectomized mice ran the similar distances (km) (SHAM=0.16 ± 0.36 km, OVX=0.036 ± 0.079 km, p > 0.05). While in the dark cycle, the SHAM mice ran more on average every hour (SHAM=0.76 ± 0.43 km, OVX=0.07 ± 0.05 km, p < 0.0001; **Figure 1C**). Total distance that was run was lower in the OVX mice ran compared to the SHAM mice (65 ± 9.2 km vs 529 ± 37.2 km, p< 0.0001; **Figure 1D**). Uterine mass has previously been used as a surrogate measure for the effects of ovarian sex hormone loss through ovariectomy, our results demonstrate that OVX mice had a lower uterine mass compared to SHAM (p<0.0001) and there was no effect of exercise (p>0.05, **Figure 1E**). Estrogen receptor content of ERα and ERB were also assessed. In the prefrontal cortex, ERα was higher in the OVX VWR mice compared to the OVX SHAM mice (p = 0.035; **Figure 1F**), while ERB was higher in the OVX VWR mice compared to all other groups (p<0.05, **Figure 1F**). In the hippocampus, there were no differences in Erα content across the groups, while ERB was higher in the SHAM VWR group compared to the SHAM SED group (p<0.05, **Figure 1G**).

### VWR maintains object recognition memory in an OVX model

**Figure 2:**
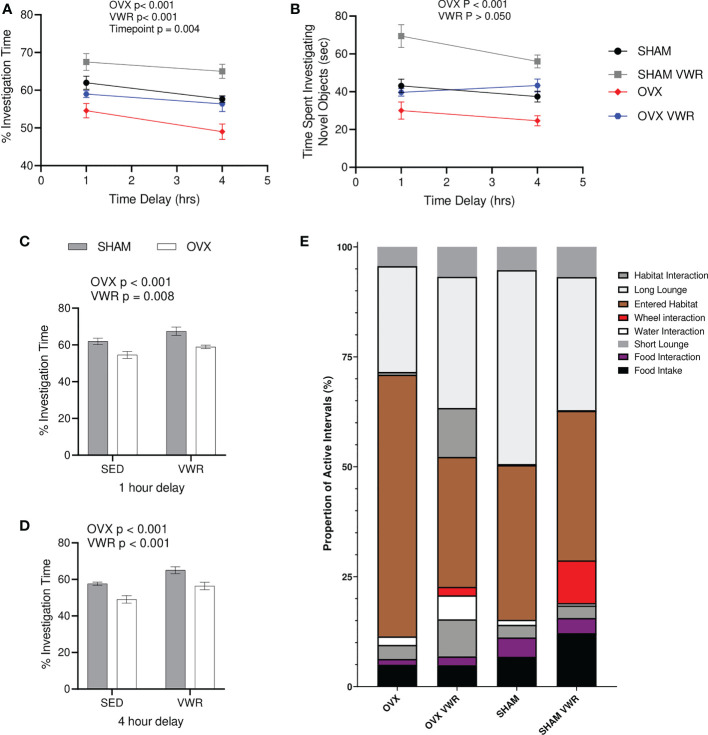
Novel Object Recognition Testing and behavioral characterization. **(A)** % investigation of novel object/total investigation time (%). **(B)** Total time spent investigating objects (s). **(C)** % investigation of novel object/total investigation time for 1-hour delay (%). **(D)** % investigation of novel object/total investigation time for 4-hour delay (%). **(E)** 24-hour breakdown of behavioral characterization. All data analyzed using a two-way ANOVA besides (B). NORT, novel object recognition testing; SED, sedentary; VWR, voluntary wheel running; OVX, ovariectomized; SHAM, sham operated. Significance is set to P ≤ 0.05; n=10 per group.

During the NORT cognitive testing for active memory recall, both the percentage of time (%) spent investigating the novel object as well as total investigation time (s) were calculated. There was a main effect of OVX showing that OVX mice had a lower percent investigation time (p < 0.0001), as well as a main effect of exercised mice having a higher percent investigation time (p < 0.0001) with no significant interaction (p > 0.05; **Figure 2A**). There was also a main effect of time (p < 0.005) as percent investigation time was lower with the 4-hour timepoint when compared with the 1-hr timepoint (p < 0.0001; **Figure 2A**). Regarding total investigation time or time spent investigating both objects, there was no main effect of exercise (p > 0.05), there was however a main effect of OVX status (p < 0.0001; **Figure 2B**). When examining the timepoints separately, the 1-hour timepoint demonstrates differences with OVX groups having lower percentage novel object investigation (p < 0.0001), while the VWR groups had a higher percentage when compared to the SED groups (p = 0.0084; **Figure 2C**). Finally, the 4-hour timepoint demonstrates similar results with the OVX having lower percentage novel object investigation (p < 0.0001) and the VWR groups having higher percentage (p = 0.0002; **Figure 2D**). Neither of the timepoints had any significant interactions (p > 0.05; **Figures 2C, D**). Behavioural phenotyping was completed where the 24hr breakdown of their behaviour and actions are shown as a stacked bar graph to more easily demonstrate any differences in behaviour in regards to time distribution (**Figure 2E**).

### Amyloidogenic markers

**Figure 3:**
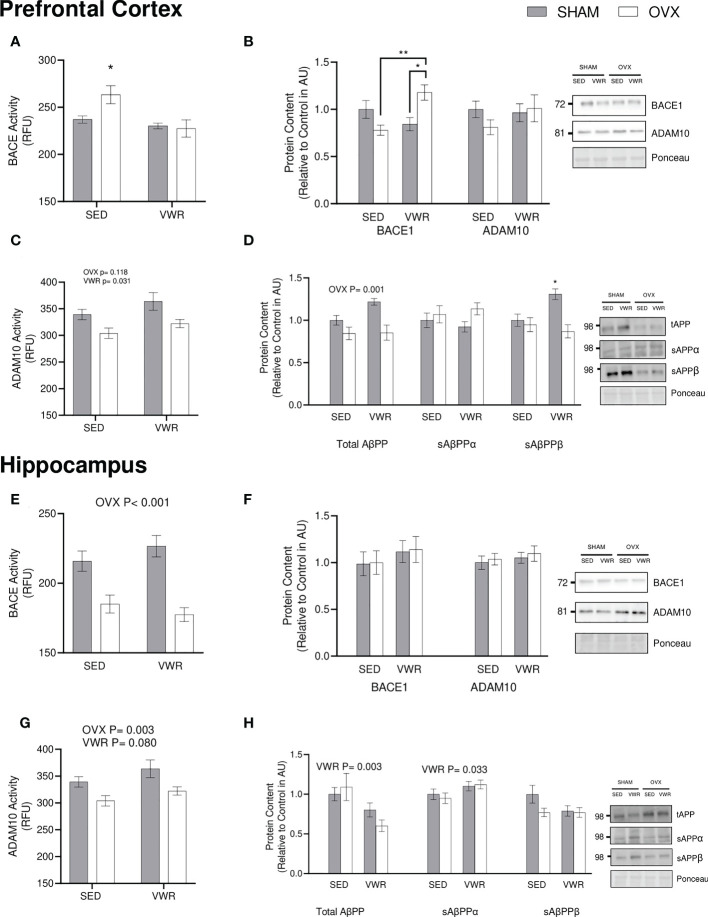
ABPP processing. **(A)** Prefrontal cortex BACE1 endpoint activity (RFU/µg total protein). **(B)** Prefrontal cortex BACE1 and ADAM10 content (AU). **(C)** Prefrontal cortex ADAM10 endpoint activity (RFU/µg total protein). **(D)** Prefrontal cortex total AβPP, sAβPPα, sABPPβ content and sAβPPα/sAβPPβ ratio (AU). **(E)** Hippocampus BACE1 endpoint activity (RFU/µg total protein). **(F)** Hippocampus BACE1 and ADAM10 content (AU). **(G)** Hippocampus ADAM10 endpoint activity (RFU/µg total protein). **(H)** Hippocampus total ABPP, sAβPPα, sAβPPβ content and sAβPPα/SaβPPβ ratio (AU). All graphs are accompanied by representative blots. All data analyzed using a two-way ANOVA. ABPP, amyloid precursor protein; SAβPPα, soluble amyloid precursor protein alpha; sAβPPβ, soluble amyloid precursor protein beta; BACE1, beta-site ABPP cleaving enzyme 1; ADAM10, A disintegrin and metalloproteinase 1; RFU, relative fluorescent units; AU, arbitrary units; SED, sedentary; VWR, voluntary wheel running; OVX, ovariectomized; SHAM, sham operated. Significance is set to *P ≤ 0.05; **P ≤ 0.005; P ≤ 0.05; n=10 per group.

As an indicator of active ABPP cleavage, endpoint BACE and ADAM10 activity were assessed. In the prefrontal cortex it was found that the sedentary OVX group had higher levels of BACE activity when compared to all other groups (p < 0.05; **Figure 3A**). In regard to ADAM10 activity in the prefrontal cortex there was no effect of OVX, however, VWR resulted in higher BACE activity (p < 0.05; **Figure 3C**). BACE1 content in the prefrontal cortex was higher in the OVX VWR group when compared the OVX SED and SHAM VWR groups however not when compared to the SHAM SED group (p < 0.05; **Figure 3B**). There were no differences in ADAM10 content between groups in the prefrontal cortex. In the hippocampus BACE activity was lower in the OVX groups (p < 0.001; **Figure 3E**) and ADAM10 activity was lower in the OVX groups (p = 0.003; **Figure 3G**) with no significant effect in the VWR groups. In regards to protein content of both enzymes, there is no significance across either protein or either group (**Figure 3F**).

Downstream markers of AβPP processing were assessed by examining total ABPP content and cleavage products of both the amyloidogenic and the nonamyloidogenic cascade (sAβPPα & sAβPPβ). In the prefrontal cortex, the OVX groups had a lower content of total ABPP (p = 0.0012; **Figure 3D**). There were no differences in sAβPPα content between groups, however sABPPB content in the SHAM VWR group was higher than any of the other groups (p < 0.05; **Figure 3D**). In the hippocampus, AβPP content was lower in VWR groups (p < 0.005; **Figure 3H**). The VWR groups also had a higher content of sAβPPα when compared to the SED groups (p < 0.05; **Figure 3H**) without any significant differences present in hippocampus sAβPPβ (p > 0.05; **Figure 3H**).

### BDNF content and signaling

**Figure 4:**
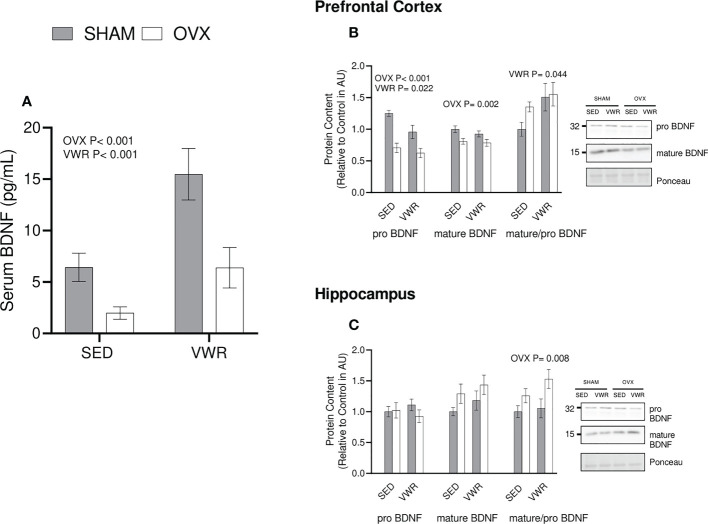
BDNF Content. **(A)** Serum BDNF as measured through ELISA (pg/mL) **(B)** Prefrontal cortex proBDNF, matureBDNF (AU) and mature/pro BDNF ratio. **(C)** Hippocampus proBDNF, matureBDNF (AU) and mature/pro BDNF ratio. All graphs are accompanied by representative blots. All data analyzed using a two-way ANOVA. BDNF, brain derived neurotrophic factor; AU, arbitrary units; SED, sedentary; VWR, voluntary wheel running; OVX, ovariectomized; SHAM, sham operated. Significance is set to P ≤ 0.05; n=10 per group.

Serum BDNF and BDNF signaling were also quantified as a potential intermediate for the observed changes in ABPP content and processing. A commercial BDNF ELISA (ChemiKine) was used to determine that there was lower serum BDNF content in the OVX groups (p = 0.0004) as well as higher content in the exercised groups (p = 0.0004; **Figure 4A**). In the prefrontal cortex there was a main effect of OVX to reduce proBDNF content (p<0.001) as well as a main effect of VWR to reduce proBDNF content (p=0.022). Mature BDNF was lower in OVX mice in the prefrontal cortex (p = 0.002). When examining the ratio of mature to proBDNF the VWR group had a significantly higher ratio (p = 0.0439; **Figure 4B**) in the PFC. In the hippocampus, there were no significant differences between all groups (p > 0.05) for pro BDNF. When looking at mature BDNF content in the hippocampus, there was no significance across all groups (p > 0.05). Finally, the ratio of mature/pro BDNF was higher in the OVX groups (p < 0.008; **Figure 4C**).

**Figure 5:**
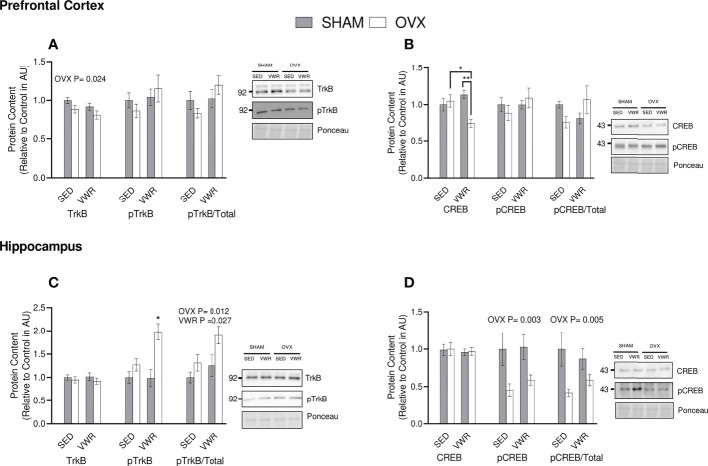
BDNF Signaling. **(A)** Prefrontal cortex total TrkB, pTrkB (AU) and pTrkB/total TrkB ratio. **(B)** Hippocampus total TrkB, pTrkB (AU) and pTrkB/total TrkB ratio. **(C)** Prefrontal cortex total CREB, pCREB (AU) and pCREB/total CREB ratio. **(D)** Hippocampus total CREB, pCREB (AU) and pCREB/total CREB ratio. All graphs are accompanied by representative blots. All data analyzed using a two-way ANOVA, TrkB, tropomyosin receptor kinase B; CREB, cAMP response element-binding protein; AU, arbitrary units; SED, sedentary; VWR, voluntary wheel running; OVX, ovariectomized; SHAM, sham operated. Significance is set to *P ≤ 0.05; **P ≤ 0.005; P ≤ 0.05; n=10 per group.

To determine the binding potential of BDNF, its main receptor, TrkB, was quantified. In the prefrontal cortex, there was a lower total TrkB content in the OVX groups (p < 0.05). There were no differences in TrkB phosphorylation status between groups (p > 0.05). Similar results were observed for the ratio of phosphorylated TrkB to total TrkB content (p > 0.05; **Figure 5A**). In the hippocampus, there were no differences across all groups in total TrkB (p > 0.05). Analyzing pTrkB demonstrated that the OVX VWR group had a higher content of pTrkB when compared to all other groups (p < 0.05). The ratio of pTrkB/TrkB was significantly higher in the VWR groups (p < 0.05) and in the groups who had been ovariectomized (p < 0.02; **Figure 5C**) showing a main effect of VWR and OVX status independently with no interaction.

To assess downstream signaling of TrkB, the content of transcription factor CREB was quantified. In the prefrontal cortex the OVX VWR group had lower CREB content compared to the OVX sedentary (p < 0.05) and SHAM VWR groups (p < 0.005). When analyzing pCREB and the pCREB/CREB ratio in the prefrontal cortex, there were no significant differences between groups (p > 0.05; **Figure 5B**). In the hippocampus, there were no differences in total CREB between groups (p > 0.05). For pCREB content, there was a main effect of OVX, where OVX mice had lower content (p < 0.05). Similar results were observed for the ratio of pCREB/CREB in the hippocampus, where the OVX groups had lower content (p = 0.005; **Figure 5D**).

## Discussion

In this study, we provide novel insight into how ovarian hormone loss alters AβPP processing as well as the beneficial effects of exercise in this model. Specifically, exercise blunted the effects of OVX on markers of amyloidogenic processing as evidenced by the reductions in BACE1 activity in the PFC and increased ADAM10 activity in the PFC and HIPP. Further, exercise resulted in an increased serum BDNF and an increase in ERα and ERβ in the PFC and ERβ in the HIPP. These biochemical adaptations were accompanied by functional changes as shown by the maintenance of active memory recall in the NORT in the OVX VWR group compared to the OVX group. Together, these findings highlight the impact that the loss of ovarian sex hormones has on markers of ABPP processing while simultaneously demonstrating the beneficial impacts of exercise on these pathological findings.

Estrogen signals by binding and activating estrogen receptors (ERα or ERβ) that can mediate genomic and/or rapid non-genomic actions. It is through these pathways that the beneficial effects of estrogen on the brain are thought to occur. There has been surprisingly little research on changes in brain ERα or ERβ content in different female rodent models and even less research examining the response to OVX or exercise (38). Our work demonstrates region specific differences in ERs in response to OVX and VWR. Most work has utilized male knockdown models of either receptor or cell culture models to investigate the role or ERs across a variety of issues (38, 39). It is thought that ERα is predominantly responsible for initiating the aforementioned connection between estrogen signaling and its impact on estrogen response elements, for example the role that estrogen plays in regulating BDNF transcription (38–40). ERα also tends to be associated with certain neuronal reward circuitry. Although it is proposed that ERB plays a significant role in sexual drive and some reward based behaviors, it is mostly associated with anxiety and neuronal branching which can ultimately impact memory (39, 40). The changes that we observed in the prefrontal cortex and hippocampus in response to OVX and VWR with ERα may ERβ may represent a mechanism by which the mice performed differently on the NORT and the observed changes in BDNF content, however more direct work is required to fully investigate this hypothesis.

Exercise is becoming known as an early intervention for AD, however, exactly how it pertains to or affects the brains of estrogen depleted models has yet to be fully elucidated. Menopause results in the cessation of the ovarian cycle resulting in a reduction in the production of ovarian hormones, such as estrogen, that are beneficial to brain health and have neuroprotective properties (41–43). Menopause has also been recently associated with higher levels of physical inactivity which drastically increase the risk of various pathologies one of which happens to be AD (44–46). The neuroprotective impacts of exercise have been well studied (3, 9, 14, 43, 47), however, our understanding of how exercise alters AβPP processing in menopausal models is currently limited. Downstream markers of BACE1 activity and content are known to increase in women experiencing menopause as well as menopausal models such as OVX (20, 21). This was seen within our study where the OVX sedentary group had higher BACE1 activity than the three other experimental groups. Our finding that VWR was able to blunt this increase in BACE1 activity in the OVX model is novel. Furthermore, these findings also correlate effectively with the functional outcomes measured, primarily the benefits that VWR had on active memory recall as shown in the NORT. The implementation of VWR was able to improve active memory recall regardless of group, demonstrating that the improvements are independent of OVX status. Exercise has also been known to increase ADAM10 which is enzymatically responsible for the non-amyloidogenic cascade and is the competing enzyme against BACE1 (3, 11, 13). SHAM groups ran significantly more than the OVX groups and had higher active memory recall paired with an increase in ADAM10 activity in both PFC and HIPP. A potential contributor to the benefits seen with exercise in this model exercise is BDNF as it was significantly increased in serum of the exercised groups. This is also reflected in the BDNF content found in the PFC as it was shown that by comparing the ratio of mature/pro BDNF, the exercised groups showed significantly higher ratio. These results demonstrate that the improvements seen functionally in the NORT as well as biochemically involved with ABPP processing may be caused by exercise-induced increases in BDNF.

Using hippocampal and prefrontal cortex tissue samples, we were able to assess impacts of ovarian sex hormone depletion and effects of exercise on cleavage products and enzymes involved with both competing pathways associated with the processing of AβPP. We effectively show that when compared to an exercised OVX group, a sedentary OVX model had a significantly higher amount of BACE1 activity in the prefrontal cortex and a significantly lower content of sAβPPα fragments in the hippocampus. These results together indicate that there are higher rates of AβPP processing occurring through the amyloidogenic pathway as opposed to the non-amyloidogenic pathway in the absence of estrogen. Both the reduction in sABPPα fragments and the increase in BACE1 activity are commonly associated with increased levels of AB peptides and subsequent plaques in patients with AD (15, 25, 48). Although we were unable to directly measure AB peptide content in our study, BACE1 is the rate-limiting enzymatic step involved with the amyloidogenic cascade and studies have demonstrated increases in AB peptide content following higher activity levels of BACE1 (3, 12, 15, 43, 49). Work from our lab has previously shown that BACE1 activity is lowered with exercise in male mice potentially explaining these compensatory effects we saw in the OVX VWR group (15).

Previous research has demonstrated a fundamental relationship between estrogen and BACE1 where it has been determined that in the presence of estrogen, BACE1 content and activity are lowered (25, 50, 51). Although in the hippocampus there were no changes in BACE1 content, BACE1 activity was lower in OVX mice with no effect of exercise. This lower hippocampal BACE1 activity paired with no OVX related change in ADAM10 activity in the hippocampus is potentially indicative of lowered rates of ABPP processing through either cascade in general. These findings are further paired with a lowering of total ABPP in the prefrontal cortex of the OVX groups. Although this was unexpected due to the significant changes seen in prefrontal cortex BACE1 activity, there are a couple of potential reasons explaining these findings. The reductions in AβPP content could be due to an increase in the degradation of AβPP overall through various means and/or a reduction in AβPP transcription and translation. ABPP could be getting degraded prior to being cleaved by either the amyloidogenic or nonamyloidogenic pathway.

The changes in biomarkers of AD tested correspond directly with the behavioural and functional differences analyzed such as those to active memory recall. As is seen in the NORT data, the introduction of voluntary exercise resulted in the OVX mice to have levels of active memory recall similar to the sedentary SHAM mice demonstrating the beneficial effects of exercise even in this menopausal model. These changes were also sustained throughout longer intertrial intervals as is demonstrated by the relationship remaining the same between the groups across both the 1 and 4 hour timepoints. While we do not have a direct mechanism for this exercise induced improvement on memory, we hypothesize that they may be due to the increased ratio of mature to proBDNF as observed in the prefrontal cortex regardless of OVX status. As BDNF transcription is known to be regulated by estrogen, we propose that exercise may be attenuating the detrimental effect of OVX on AD biomarkers and memory by recovering BDNF content and signaling.

## Conclusions

This study provides novel insight into the beneficial effects of exercise on active memory recall as well as neurophysiological markers associated with AD in an ovarian sex hormone depleted model. The majority of AD related research uses male models despite females being disproportionally impacted by AD. The results of this study contribute to our mechanistic understanding of ovarian hormone loss and the potential that exercise has in improving overall female brain health. Our work demonstrates the beneficial effects of physical activity on maintaining active memory recall and blunting/preventing alterations in ABPP processing that are present in an OVX model. This study advances our current understanding potentially helping us better build towards answering why females have higher risks of developing AD. It further highlights the importance of promoting physical activity and exercise to lower the difference in AD prevalence between the sexes.

## Data availability statement

The raw data supporting the conclusions of this article will be made available by the authors, without undue reservation.

## Ethics statement

The animal study was reviewed and approved by Brock University Animal Care Committee (AUP: No. 17-12-01) and followed all guidelines of the Canadian Council on Animal Care.

## Author contributions

RM and AM conceived and designed the research. AM, MF, and JS-M performed the research and acquired the data, AM and RM analyzed and interpreted the data. All authors contributed to the article and approved the submitted version.
